# No Differences in Urine Bisphenol A Concentrations between Subjects Categorized with Normal Cognitive Function and Mild Cognitive Impairment Based on Montreal Cognitive Assessment Scores

**DOI:** 10.3390/metabo14050271

**Published:** 2024-05-08

**Authors:** Małgorzata Jamka, Szymon Kurek, Aleksandra Makarewicz-Bukowska, Anna Miśkiewicz-Chotnicka, Maria Wasiewicz-Gajdzis, Jarosław Walkowiak

**Affiliations:** Department of Pediatric Gastroenterology and Metabolic Diseases, Poznan University of Medical Sciences, Szpitalna Str. 27/33, 60-572 Poznan, Poland; mjamka@ump.edu.pl (M.J.); skurek@ump.edu.pl (S.K.); amakarewicz@ump.edu.pl (A.M.-B.); chotnicka@ump.edu.pl (A.M.-C.); 65284@student.ump.edu.pl (M.W.-G.)

**Keywords:** cognition, cognitive dysfunction, bisphenol A

## Abstract

A link between bisphenol A (BPA) exposure and cognitive disorders has been suggested. However, the differences in BPA concentrations between subjects with and without cognitive impairment have not been analysed. Therefore, this observational study aimed to compare urine BPA levels in subjects with normal cognitive function (NCF) and mild cognitive impairment (MCI). A total of 89 MCI subjects and 89 well-matched NCF individuals were included in this study. Cognitive functions were assessed using the Montreal Cognitive Assessment (MOCA) scale. Urine BPA concentrations were evaluated by gas chromatography–mass spectrometry and adjusted for creatinine levels. Moreover, anthropometric parameters, body composition, sociodemographic factors, and physical activity were also assessed. Creatinine-adjusted urine BPA levels did not differ between the NCF and MCI groups (1.8 (1.4–2.7) vs. 2.2 (1.4–3.6) µg/g creatinine, *p* = 0.1528). However, there were significant differences in MOCA results between groups when the study population was divided into tertiles according to BPA concentrations (*p* = 0.0325). Nevertheless, multivariate logistic regression demonstrated that only education levels were independently associated with MCI. In conclusion, urine BPA levels are not significantly different between subjects with MCI and NCF, but these findings need to be confirmed in further studies.

## 1. Introduction

The global elderly population is projected to increase to 1.4 billion by 2030, 2.1 billion by 2050, and 3.1 billion by 2100 [1]. A significant repercussion of ageing is the onset of mild cognitive impairment (MCI), a condition prevalent in about 20% of individuals over 50 [2]. MCI manifests as a decline in cognitive abilities, including memory, orientation, planning, decision-making, and comprehension, but does not significantly impact daily social or occupational functions [3,4]. It can affect language, visuospatial skills, attention, and executive functions, sometimes involving memory loss [5]. The condition is associated with an increased risk of developing dementia, with an estimated 5% to 10% of MCI patients progressing to dementia annually [6]. Prompt diagnosis could help in slowing the advancement of the disease, so identifying risk factors leading to MCI and its evolution into more severe neurocognitive disorders is crucial. Research has pinpointed several causative factors such as inadequate physical activity, alcohol consumption, smoking, substandard diet, social, environmental, and other factors [7]. Moreover, it was suggested that exposure to bisphenol A (BPA) may affect cognitive functions [8].

BPA (4,4-(propane-2-ylidene)diphenol) is widely utilised to produce polycarbonate plastics and epoxy resins, appearing in various consumer products, including food containers, packaging materials, cans, bottles, and plastic bags. Dietary sources are typically the primary route of BPA exposure [9], with BPA being rapidly absorbed in the gastrointestinal tract and almost entirely metabolised in the liver through conjugation with glucuronic acid and its remnants eliminated via urine in less than 24 h. Thus, BPA exposure can be assessed in urine [10,11], with approximately 90% of urine samples testing positive for BPA [12]. As an endocrine-disrupting chemical, BPA’s potential link to various health issues has been investigated [13,14,15], suggesting a correlation between BPA exposure and neurological disorders and cognitive impairment [16,17,18]. Animal studies showed that BPA could impair certain brain functions, including memory and learning [19,20,21]. Moreover, prenatal BPA exposure has been linked to neurobehavioral challenges in children [22,23], with maternal exposure affecting brain regions such as the postcentral gyrus, the opercular part of the inferior frontal gyrus, and the superior occipital gyrus [24]. However, there is a lack of studies assessing BPA levels in individuals with and without cognitive impairments.

Therefore, the purpose of this study was to assess urine bisphenol A levels in subjects with MCI and normal cognitive function (NCF).

## 2. Materials and Methods

### 2.1. Study Design and Oversight

This observational study was approved by the Ethical Committee of the Poznan University of Medical Sciences (protocol no.: 249/22, date of approval: 10 March 2022). The study was performed and reported according to the Strengthening the Reporting of Observational Studies in Epidemiology (STROBE) guidelines [25] (see Supplementary Materials, Table S1) and the Declaration of Helsinki [26]. All participants provided written informed consent.

### 2.2. Study Population

In this study, we employed a cross-sectional study design to compare individuals with MCI and NCF [27]. Recruitment and enrolment occurred over approximately 14 months (from July 2021 to August 2022) through the distribution of leaflets and posters, posting of recruitment information on the internet, and invitations to participate in the survey sent to various companies, associations, and institutions located in Poznań (Greater Poland Voivodeship) and the surrounding area. The qualification visits were performed by physicians in the Department of Pediatric Gastroenterology and Metabolic Diseases, Poznan University of Medical Sciences. Participants were eligible if they were 50 to 70 years old and scored 19 to 26 points (the MCI group) or 27 to 30 points (the NCF group) on the Montreal Cognitive Assessment (MOCA) scale. Among the criteria for exclusion were a history of depression treatment, obtaining in the Hamilton Depression Rating scale (HAM-D) test scores of more than 13 points, cognitive enhancement drug or psychotropic medication usage, significant alcohol consumption, substance abuse disorders, mental health conditions, Parkinson’s disease, Alzheimer’s disease, dementia, anaemia, diabetes of at least ten years duration, severe chronic kidney and hepatic disorders, recent chemotherapy or radiotherapy for cancer within the past five years, stroke, seizures within the past two years, significant head injury, hypothyroidism with abnormal thyrotropic hormone levels, other severe chronic illnesses precluding participation, and conditions such as blindness, deafness, communication challenges, or other disabilities that might impede involvement in the study.

### 2.3. Montreal Cognitive Assessment Scale

The MOCA questionnaire was completed during the qualification visit to assign the participants to the MCI and NCF groups. The assessment was performed by physicians who had obtained certificates for MOCA administration. A MOCA score < 19 points indicated dementia, scores of 19-26 points were classified as MCI, and scores > 26 points suggested NCF [28].

### 2.4. Hamilton Depression Rating Scale

The HAM-D questionnaire was also applied to exclude subjects with active depression. A HAM-D score ≥ 23 points indicated very severe depression, 18–22 points severe depression, 14–18 points moderate depression, 8–13 points mild depression, and <7 points no depressive symptoms [29,30].

### 2.5. International Physical Activity Questionnaires

The long Polish version of the International Physical Activity Questionnaire (IPAQ) was used to assess self-reported physical activity. This 27-item survey assessed the duration, frequency, and intensity of physical activity performed within the last seven days. Four different types of physical activity were assessed: job- and transport-related activity, domestic activity, and sedentary time on a usual weekday and weekend day. Only activities with a duration of at least ten minutes were registered to calculate total physical activity [31].

### 2.6. Anthropometric Parameters and Body Composition

Trained personnel measured weight and height using a calibrated scale with a stadiometer (Radwag, WPT 100/200 OW, Radom, Poland) to calculate body mass index (BMI) [32]. The measurements were performed without shoes and in underwear. The waist circumference was measured at the umbilicus in the standing position on bare skin using non-stretchable tape (Seca, Hamburg, Germany) [33]. Body composition measurements were conducted through dual-energy X-ray absorptiometry (DEXA), utilising the Hologic Discovery DEXA system (Bedford, MA, USA). The analysis focused on evaluating lean mass parameters, such as the appendicular lean mass index and the lean mass index [34].

### 2.7. Sociodemographic Questionnaire

The subjects’ place of residence, relationship status, education level, socio-occupational status, financial situation, and smoking and alcohol habits, as well as the use of selected groups of medications, were evaluated using sociodemographic questionnaires.

### 2.8. Bisphenol A Levels

The study participants were asked to provide a fasting urine sample, which was frozen at −80 °C and stored until the creatinine and total BPA levels were analysed. Creatinine levels were assessed using enzymatic methods to adjust total BPA levels to creatinine in urine. Total (free plus conjugated species) BPA concentrations were measured with gas chromatography–mass spectrometry (GC-MS). Measurements were performed with an Agilent Technologies 7890A gas chromatography system connected to a 5975C VL MSD mass spectrometer with a three-axis detector (Agilent Technologies, Waldbronn, Germany). Briefly, 0.5 mL of the urine sample was mixed with 50 μL of internal standard (deuterated BPA-d16 at 500 ng/mL) and 30 μL of acetate buffer (pH = 5.5) before the addition of 30 μL of β-glucuronidase/sulfatase (Helix pomatia, diluted 10× to 100,000 U/mL in acetate buffer). This mixture was incubated at 37 °C for three hours, and BPA was extracted with 3 × 4 mL of a dichloromethane/hexane (1:1) mixture. The extract was evaporated to dryness under a stream of nitrogen and silylated with the addition of 100 μL of N,O-bis(trimethylsilyl)trifluoroacetamide (BSTFA)/pyridine (1:1) at 80 °C for 30 min. The samples were placed in chromatographic vials with an insert for GC-MS analysis. All reagents and chemicals used to measure total BPA were purchased from Merck Life Science Sp. z. o. o., Poland (an affiliate of Merck KGaA, Darmstadt, Germany). The following GC settings were used: the oven temperature was held at 90 °C for one minute, with an increase of 10 °C/minute to 240 °C, held for two minutes, then increased by 20 °C/minute to 310 °C, and held for three minutes. The carrier gas was helium maintained at a constant pressure mode with a flow rate of 1 mL/minute at 90 °C. The HP-5MS column measured 30 m × 0.25 mm × 0.25 μm. The injector port temperature was set at 295 °C in splitless mode. The MS detector used electron ionisation with the ion source temperature at 230 °C and the quadrupole temperature at 150 °C.

### 2.9. Minimum Sample Size Calculation

The power calculation was performed using preliminary findings from a pilot study and G*Power 3.1 software from the University of Kiel, Germany. Assuming an expected dropout rate of 20%, enrolling 166 participants (83 in each group) would ensure the study has over 80% statistical power to identify a meaningful difference of 0.5 μg total urine BPA/g creatinine (assuming a standard deviation of 35% of the mean) between the groups at a significance level of 0.05.

### 2.10. Statistical Analysis

Statistical analyses were calculated using Statistica 13.0 (TIBCO Software Inc., Palo Alto, CA, USA) and PQstat 1.8.4 (PQStst Software Poznań/Plewiska, Poland) software. The Shapiro–Wilk test was used to check if the data were normally distributed. The Mann–Whitney test was applied to compare MCI and NCF individuals. Moreover, the Kruskal–Wallis test and the Jonckheere–Terpstra trend test were performed to compare subjects divided into tertiles according to urine BPA levels. The Fisher exact test, Pearson’s Chi-squared test, and the Cochran–Armitage trend test were applied to evaluate categorical variables. In addition, logistic regression was performed to determine the variables predicting the occurrence of MCI, while linear regression analysis was performed to analyse the relationship between urine BPA concentration and selected variables. The variables from the univariate regression analysis with a significance level of *p* < 0.1 were included in a multivariate regression model. The propensity score matching 1:1 method was used to match subjects from the MCI group with those from the NCF group based on age, sex, and BMI. All data are presented as medians and interquartile ranges or as counts and percentages. Analysis was used at an α-level of 0.05 to identify statistical significance.

## 3. Results

### 3.1. Study Workflow

**Figure 1:**
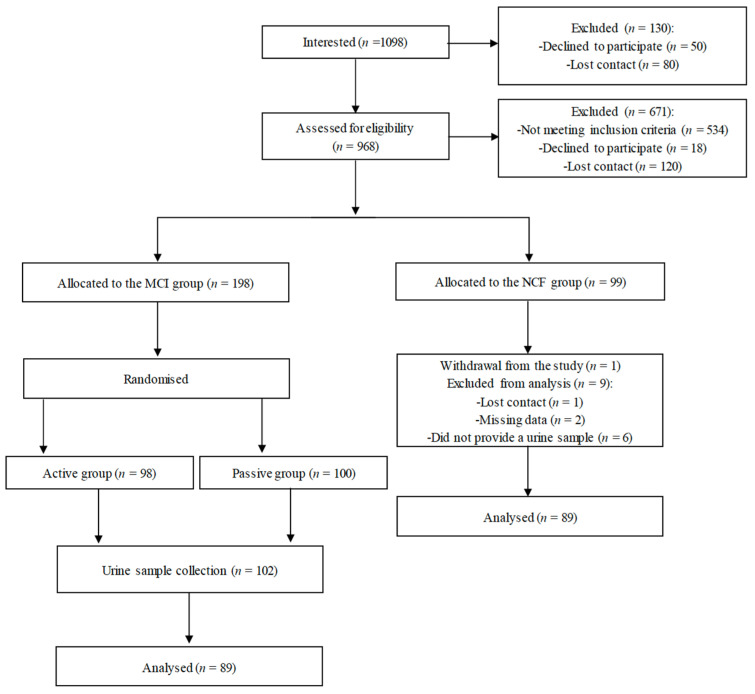
Study flow chart.

Figure 1 illustrates the workflow of this study. As described earlier [27], approximately 1000 individuals were interested in participating, of which 969 were eligible. Of these, 99 were initially placed in the NCF group, but 1 participant opted out of the study, and 9 were excluded from the analysis due to various reasons, so the NCF group ultimately included 89 participants. Additionally, among the participants with MCI who were part of the randomised controlled trial [35] and collected a urine sample, 89 were selected to match the NCF group by age, sex, and BMI for inclusion in this analysis. The baseline characteristics of the participants, such as sex, age, body measurements, HAM-D score, total physical activity, and sociodemographic aspects, are detailed in Table 1 and Table 2, showing no significant differences between the groups.

### 3.2. Comparison of Bisphenol A Levels between the Normal Cognitive Function and Mild Cognitive Impairment Groups

**Figure 2:**
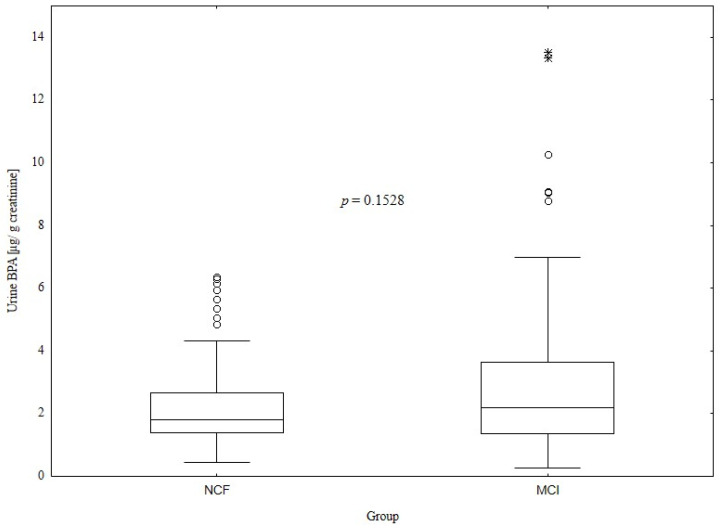
Comparison of urine BPA levels between the NCF and MCI groups (1.8 (1.4–2.7) vs. 2.2 (1.4–3.6) µg/g creatinine, *p* = 0.1528).

Figure 2 presents the comparison of adjusted urine BPA levels between subjects with NCF and MCI, showing no significant differences between the groups (NCF vs. MCI: 1.8 (1.4—2.7) vs. 2.2 (1.4—3.6) µg/g creatinine, *p* = 0.1528).

### 3.3. Comparison of Study Population According to Urine Bisphenol A Tertiles

**Figure 3:**
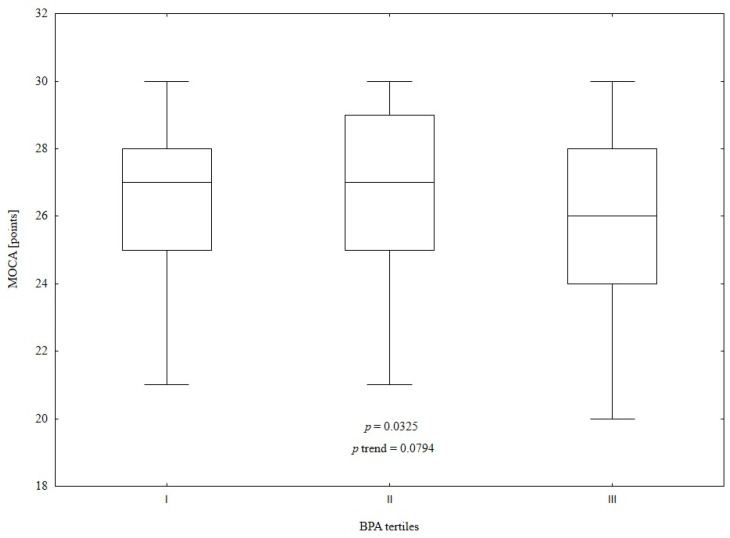
Comparison of MOCA scores between BPA tertiles (I vs. II vs. III: 27 (27–28) vs. 27 (25–29) vs. 26 (24–28) points, *p* = 0.0325, *p* trend = 0.0794). Post hoc test results: I vs. II: *p* = 1.0000, I vs. III: *p* = 0.2588, II vs. III: *p* = 0.0330.

We divided the study population into tertiles according to urine BPA concentrations (see Supplementary Materials, Table S2) and found significant differences in MOCA results (*p* = 0.0325) between groups (see Figure 3). Post hoc tests showed that subjects from tertile II had obtained statistically significantly higher MOCA scores than subjects from tertile III (*p* = 0.0330). However, the trend test did not find a significant trend between groups in the MOCA results.

### 3.4. Associations between Urine Bisphenol A Concentrations and Predictive Variables

Univariate linear regression showed that age (*p* = 0.0111) and socio-occupational status (*p* = 0.0428) were associated with urine BPA concentrations (see Table 3). However, multivariate analysis showed that none of these factors were associated with urine BPA concentrations (see Table 4).

### 3.5. Predictive Factors of Mild Cognitive Impairment Prevalence

Logistic regression was performed to identify factors that might predict the probability of MCI. Univariate logistic regression showed that the total physical activity (*p* = 0.0437) and education levels (*p* = 0.0184) were determinants of MCI (see Table 5). These factors were included in multivariate logistic regression analysis, which showed that only education levels (*p* = 0.0220) were independently associated with MCI development (see Table 6).

## 4. Discussion

The present study revealed no significant difference in creatinine-adjusted urine BPA levels between subjects with NCF and MCI, suggesting that BPA exposure may not directly differentiate these groups. However, when the study population was divided into tertiles according to BPA concentrations, there were significant differences between MOCA scores.

In our study, BPA was detected in all analyzed samples with a median urine BPA concentration of 2.1 µg/g creatinine, which is slightly higher than previously reported [36,37,38]. Park et al. [38] reported a mean urine BPA concentration of 2.01 μg/g creatinine in 2044 Korean participants across different age groups, collecting 12-h urine samples and using high-performance liquid chromatography–tandem mass spectrometry. They did not observe any differences between men and women but noted a positive association between BPA levels and age. The authors also performed a literature search to assess differences in urine BPA levels between different countries and found higher urine BPA levels in the European population than in people from other countries. The researchers speculated that these differences in BPA levels may result from variations in canned food intake worldwide [39]. Calafat et al. [36] analysed BPA concentrations in 394 adult participants of the Third National Health and Nutrition Examination Survey study using the isotope dilution GC-MS method and spot-urine samples collected at various times throughout the day. BPA levels were detected in 95% of the analysed samples, with a median BPA concentration of 1.32 µg/g creatinine. In addition, Koch et al. [37] investigated BPA levels in 24-h urine and plasma samples from the German Environmental Specimen Bank spanning from 1995 to 2009 by high-performance liquid chromatography coupled to isotope dilution tandem mass spectrometry methods, and BPA was present in more than 96% of samples. The average total BPA concentration was slightly lower than that observed in our study (1.81 µg/g creatinine). Also, a recent meta-analysis, which included 15 studies and 28,353 individuals, reported that BPA was present in 90% of samples, with pooled BPA concentrations of 1.76 μg/g creatinine. Moreover, this meta-analysis showed that factors such as age, sex, residence of study participants, and the measurement method did not affect urine BPA levels [40]. In our study, univariate linear regression showed that age and socio-occupational status were associated with urine BPA concentrations. However, these factors were not associated with creatinine-adjusted urine BPA concentrations in multivariate analysis. Nevertheless, it was previously suggested that men might be more sensitive to BPA than women due to the greater binding to oestrogen receptors [41]. Furthermore, Zhang et al. [42] reported differences in urine BPA levels between men and women and subjects of different ages. The predominance of women in our study population may partly explain why sex was not identified as a factor influencing BPA levels in our analysis. However, we speculate that different levels of BPA exposure from the diet and other sources might partly explain differences in urine BPA levels and factors affecting BPA concentrations observed in various studies [43].

The negative effect of BPA on human health is well known. Bao et al. [14] demonstrated that higher exposure to BPA may increase all-cause mortality in a US population. Moon et al. [44], in their meta-analysis, showed that urine BPA levels are associated with the prevalence of cardiovascular diseases. BPA exposure is also related to an increased risk of type 2 diabetes mellitus [45]. Moreover, there is a relationship between BPA concentrations and obesity [46], as well as polycystic ovary syndrome [47]. It has also been suggested that BPA exposure may affect cognitive function [8]. However, our study did not detect differences in creatinine-adjusted urine BPA levels between MCI and NCF subjects, which may be associated with the small sample size and measuring the BPA concentration only in a single urine sample. Nevertheless, subjects in tertile II of BPA levels had statistically significantly higher MOCA scores than subjects from the highest tertile.

Interestingly, our analysis revealed greater variability in BPA levels among participants diagnosed with MCI compared to those with NCF. Several factors could contribute to this variability, including the subjects’ lifestyle behaviours and their health status. Differences in dietary habits [27] and physical activity levels [48] could account for this variability. Moreover, there may be physiological differences in how BPA is metabolised or excreted in individuals with MCI compared to those without cognitive impairments. Changes in kidney function, which are sometimes associated with cognitive decline, could also influence BPA levels [49].

This is the first study that compared BPA levels between NCF and MCI participants. Previous studies that assessed the effect of BPA on cognition focused on the assessment of the impact of maternal exposure on foetus neurodevelopment or evaluated the effect of BPA exposure in children [22,50,51]. Braun et al. [51] observed that prenatal BPA exposure had a more negative effect on cognitive function in boys than in girls. BPA concentrations affected behaviours involving internalisation and somatisation and were associated with poorer working memory. Similarly, Rodríguez-Carrillo et al. [50] showed that BPA concentrations standardised to creatinine levels were associated with a higher risk of poorer working memory scores in school-age boys. Huang et al. [22] discovered the different effects of maternal BPA exposure in girls and boys. Higher BPA levels in girls were associated with enhanced dangers of self-control inhibition, developing metacognition issues, behavioural challenges, difficulties in peer relationships, elevated total difficulties score, and increased impact factor score, whereas higher prenatal BPA levels in boys increased the risk of behavioural problems. In addition, prenatal BPA exposure was also associated with the prevalence of attention deficit hyperactivity disorder (ADHD) in children aged three years. An association between BPA levels and ADHD prevalence was also confirmed by Gok et al. [18], who reported that subjects with ADHD had significantly higher BPA levels than participants without ADHD. However, in this study, BPA concentrations did not correlate with cognitive function.

Several mechanisms have been proposed to explain the potential negative impact of BPA on cognitive functions [52,53]. Both in vivo and in vitro studies have demonstrated the effect of BPA on apoptosis, oxidative stress, inflammation, mitochondrial dysfunction, and endoplasmic reticulum stress [54,55,56,57,58]. Moreover, the detrimental effect of BPA on myelination processes and neuronal integrity has been reported in both cell and animal studies [59,60]. It has also been shown that BPA might modulate neurotransmitter levels [61,62,63,64]. Furthermore, BPA’s neurotoxicity has been associated with inhibition of neurogenesis [65] and reduced synaptic plasticity in rats [66]. Additional research has identified decreased axon length in zebrafish models [57], microglial DNA damage, and astrogliosis as responses to BPA exposure in explant models [67]. Liu et al. [68] and Li et al. [69], in human and animal studies, suggested that BPA may disrupt the homeostasis of energy metabolism and insulin signalling pathways, thereby impairing cognitive functions. However, another in vitro study noted that BPA affects intracellular Ca^2+^ balance [70]. Furthermore, incubation of a human neuroblastoma cell line with BPA increased levels of β-amyloid and tau proteins, which are linked to the pathogenesis of Alzheimer’s disease [71].

Herein, we also performed logistic regression to identify independent determinants of MCI prevalence. The univariate logistic regression revealed that total physical activity and education impact MCI development. However, despite the suggestion that physical activity may be a protective factor for MCI [72,73,74], the multivariate logistic regression analysis demonstrated that only lower education levels were associated with higher MCI prevalence. Our findings are in line with those previously reported [75,76,77]. Bai et al. [75] reported that MCI prevalence increased with lower education levels, while Xue et al. [76] found that higher education levels may predict revision from MCI to NCF. Moreover, Vadikolias et al. [77] indicated that education was associated with results obtained in denominating entities, definitions, linguistic constructs, ability to name upon confrontation, or phonetic assistance. In addition, Xu et al. [78], in their meta-analysis, showed that the risk of dementia was lowered by 7% for each additional year of education.

Our results have some potential clinical implications. First, the finding that a higher level of education may reduce the risk of developing cognitive disorders indicates that subjects with lower educational levels could benefit from earlier and more frequent cognitive assessments to detect potential decline more promptly. Additionally, although physical activity was not independently associated with MCI in our multivariate regression analysis, its protective role in cognitive health, as confirmed in several studies [72,73,74], suggests that increasing physical activity could be a beneficial strategy to prevent cognitive decline. Furthermore, as the relationship between BPA and cognitive decline was not definitively established in our study, it is important to consider other factors that may contribute to the development of MCI.

This study is among the initial investigations to compare urine BPA levels between individuals with NCF and MCI. The study’s strengths include the implementation of stringent and well-defined criteria for inclusion and exclusion, along with the application of propensity score matching to ensure that both groups are comparable in terms of age, sex, and BMI, as well as the adjustment of urine BPA levels for creatinine concentrations.

However, this study has some limitations, including categorising participants into the MCI and NCF groups based solely on MOCA test results. Moreover, we categorised participants into groups based on a single cognitive function assessment and did not compare it against peers of the same sex, age, and education levels. Since the MOCA assessment was also not repeated for study participants and we did not evaluate symptoms of subjective cognitive decline, there could potentially be a risk of inappropriately categorising subjects into study groups. It should also be highlighted that most individuals included in our study had higher education levels, lived in the city, were professionally active, and had moderately good health status. Therefore, our results should be generalised with caution to other populations, including subjects with lower education levels or those who live in rural environments, as BPA exposure in these individuals might differ. Another limitation of this study is relying on a single urine sample to measure BPA levels, which may not accurately reflect long-life exposure. Additionally, no assessment of BPA in blood samples was conducted. Furthermore, we did not evaluate BPA exposure directly and only measured total urine BPA levels without analysing the concentrations of free and conjugated BPA. Assessing conjugated BPA levels could mitigate potential BPA contamination during the collection, storage, and analysis of urine samples, as this form is produced within the human body. However, analysis of the conjugated form may be difficult, and therefore, measurement of total BPA concentrations is recommended to assess BPA exposure [79]. Moreover, the hydration levels of the study participants were not assessed, and hydration might affect urine creatinine levels [80].

## 5. Conclusions

In conclusion, there were no significant differences in urine BPA levels in subjects with MCI from those in NCF individuals, but further larger studies are required to confirm the impact of BPA on neurocognitive functions. Future studies should compare the incidence of cognitive disorders between individuals with low and high BPA exposure to better understand the relationship between BPA exposure and cognitive decline. Furthermore, the short-term and long-term exposures could be compared.

## Figures and Tables

**Table 1:** Baseline characteristics of the study population.

	Total (*n* = 178)	NCF (*n* = 89)	MCI (*n* = 89)	*p*
	Median (Q1–Q3)			
Age [years]	58 (54–62)	56 (54–61)	59 (54–62)	0.5385
Weight [kg]	75.50 (64.40–87.50)	76.0 (64.4–91.0)	75.00 (64.50–84.60)	0.4750
BMI [kg/m^2^]	27.07 (23.88–30.39)	26.70 (23.53–30.75)	27.12 (24.56–30.00)	0.6605
Waist circumference [cm]	90 (80–100)	89 (80–102)	93 (80–100)	0.8728
LMI [kg/m^2^]	16.05 (14.20–18.00)	16.00 (14.10–18.30)	16.10 (14.40–17.70)	0.8958
ALMI [kg/m^2^]	6.73 (5.87–7.88)	6.91 (5.85–8.00)	6.66 (5.91–7.61)	0.5326
Total physical activity [min/day]	99 (56–155)	105 (59–163)	81 (54–150)	0.1259
HAM-D [points]	3 (1–6)	4 (1–6)	3 (1–5)	0.8846

ALMI—appendicular lean mass index; BMI—body mass index; HAM-D—Hamilton depression rating scale; LMI—lean mass index; MCI—mild cognitive impairment; NCF—normal cognitive function.

**Table 2:** Comparison of sociodemographic parameters between subjects with NCF and MCI.

		Total (*n* = 178)	NCF (*n* = 89)	MCI (*n* = 89)	*p*
		*n* (%)			
Sex	Women	130 (73.0%)	64 (71.9%)	66 (74.2%)	0.8660
	Men	48 (27.0%)	25 (28.1%)	23 (25.8%)	
Place of residence	Village	29 (16.3%)	13 (14.6%)	16 (18.0%)	0.8504
	City < 50.000 inhabitants	14 (7.9%)	6 (6.7%)	8 (9.0%)	
	City of 50.000–500.000 inhabitants	15 (8.4%)	8 (9.0%)	7 (7.9%)	
	City > 500.000 inhabitants	120 (67.4%)	62 (69.7%)	58 (65.2%)	
Relationship status	Formal/informal relationship	137 (77.0%)	72 (80.9%)	65 (73.0%)	0.2631
	Single	40 (22.5%)	17 (19.1%)	23 (25.8%)	
	No information	1 (0.6%)	0 (0.0%)	1 (1.1%)	
Education	Primary	2 (1.1%)	0 (0.0%)	2 (2.2%)	0.0562
	Vocational	6 (3.4%)	1 (1.1%)	5 (5.6%)	
	Secondary	29 (16.3%)	11 (12.4%)	18 (20.2%)	
	High	141 (79.2%)	77 (86.5%)	64 (71.9%)	
Socio-occupational status	Employed	144 (80.9%)	74 (83.1%)	70 (78.7%)	0.7367
	Unemployed	2 (1.1%)	1 (1.1%)	1 (1.1%)	
	Pensioner	32 (18.0%)	14 (15.7%)	18 (20.2%)	
Financial situation	Very good	16 (9.0%)	7 (7.9%)	9 (10.1%)	0.5156
	Good	121 (66.0%)	60 (67.4%)	61 (68.5%)	
	Mediocre	39 (21.9%)	20 (22.5%)	19 (21.3%)	
	Bad	2 (1.1%)	2 (2.2%)	0 (0.0%)	
Formerly smoking	Yes	70 (39.3%)	31 (34.8%)	39 (43.8%)	0.2827
	No	108 (60.7%)	58 (65.2%)	50 (56.2%)	
Currently smoking	Yes	17 (9.6%)	7 (7.9%)	10 (11.2%)	0.6112
	No	161 (90.4%)	82 (92.1%)	79 (88.8%)	
Alcohol consumption	Once a day	4 (2.2%)	3 (3.4%)	1 (1.1%)	0.3085
	Several times a week	22 (12.4%)	15 (16.9%)	7 (7.9%)	
	Once a week	45 (25.3%)	21 (23.6%)	24 (27.0%)	
	1–3 times a month	71 (39.9%)	32 (36.0%)	39 (43.8%)	
	Never	36 (20.2%)	18 (20.2%)	18 (20.2%)	
Antihypertensive drugs	Yes	50 (28.1%)	23 (25.8%)	27 (30.3%)	0.6171
	No	128 (71.9%)	66 (74.2%)	62 (69.7%)	
Hypolipemic drugs	Yes	17 (9.6%)	7 (7.9%)	10 (11.2%)	0.6112
	No	161 (90.4%)	82 (92.1%)	79 (88.8%)	
Hypoglycaemic drugs	Yes	8 (4.5%)	2 (2.2%)	6 (6.7%)	0.2778
	No	170 (95.5%)	87 (97.8%)	83 (93.3%)	
Hypothyroidism drugs	Yes	29 (16.3%)	17 (19.1%)	12 (13.5%)	0.4173
	No	149 (83.7%)	72 (80.9%)	77 (86.5%)	

MCI—mild cognitive impairment; NCF—normal cognitive function.

**Table 3:** Univariate linear regression analysis assessing the relationship between urine BPA [µg/g creatinine] and selected variables in the total study population.

	β	SE	t	*p*
Age [years]	0.1900	0.0740	2.5669	0.0111
BMI [kg/m^2^]	−0.0582	0.0753	−0.7734	0.4403
LMI [kg/m^2^]	−0.1147	0.0748	−1.5340	0.1268
ALMI [kg/m^2^]	−0.0373	0.0753	−0.4955	0.6208
Total physical activity [min/day]	0.0553	0.0753	0.7351	0.4633
MOCA [points]	−0.0891	0.0751	−1.1870	0.2368
HAM-D [points]	0.0294	0.0753	0.3902	0.6969
Sex ^1^	0.0955	0.0750	1.2724	0.2049
Place of residence ^2^	−0.0558	0.0753	−0.7416	0.4593
Relationship status ^3^	−0.0738	0.0754	−0.9791	0.3289
Education ^4^	−0.0655	0.0752	−0.8713	0.3848
Socio-occupational status ^5^	−0.1520	0.0745	−2.0401	0.0428
Financial situation ^6^	−0.0989	0.0750	−1.3185	0.1891
Formerly smoking ^7^	0.1152	0.0749	1.5386	0.1257
Currently smoking ^7^	0.0632	0.0752	0.8396	0.4023
Alcohol consumption ^7^	0.0708	0.0752	0.9414	0.3478
Antihypertensive drugs ^7^	−0.0161	0.0754	−0.2131	0.8315
Hypolipemic drugs ^7^	−0.0646	0.0752	−0.8590	0.3915
Hypoglycaemic drugs ^7^	0.0059	0.0754	0.0780	0.9379
Hypothyroidism drugs ^7^	0.0109	0.0754	0.1446	0.8852

^1^ Women vs. men; ^2^ village vs. city; ^3^ in relationship vs. single; ^4^ high vs. other; ^5^ employed vs. other; ^6^ very good + good vs. other; ^7^ yes vs. no. ALMI—appendicular lean mass index; BMI—body mass index; BPA—bisphenol A; HAM-D—Hamilton depression rating scale; HDL-C—high-density lipoprotein cholesterol; hsCRP—high-sensitivity C reactive protein; LDL—low-density lipoprotein cholesterol; LMI—lean mass index; MOCA—Montreal cognitive assessment scale; SE—standard error; TC—total cholesterol; TG—triglycerides.

**Table 4:** Multivariate linear regression analysis assessing the relationship between urine BPA corrected [µg/g creatinine] and selected variables in the total study population.

	β	SE	t	*p*
Age [years]	0.1532	0.0905	1.6921	0.0924
Socio-occupational status ^1^	−0.0640	0.0905	−0.7064	0.4809

^1^ Employed vs. other. BPA—bisphenol A; SE—standard error.

**Table 5:** Unadjusted results of logistic regression analysis predicting the probability of MCI.

	OR	95% CI	*p*
Age [years]	1.016	0.961–1.075	0.5690
BMI [kg/m^2^]	0.998	0.945–1.055	0.9537
LMI [kg/m^2^]	0.966	0.862–1.081	0.5444
ALMI [kg/m^2^]	1.000	0.997–1.003	0.9004
Total physical activity [min/day]	0.996	0.993–1.000	0.0437
HAM-D [points]	0.989	0.888–1.102	0.8474
Urine BPA [µg/g creatinine]	1.032	0.946–1.127	0.4764
Sex ^1^	1.121	0.578–2.174	0.7356
Place of residence ^2^	1.281	0.576–2.850	0.5432
Relationship status ^3^	0.667	0.328–1.359	0.2647
Education ^4^	0.399	0.186–0.857	0.0184
Socio-occupational status ^5^	1.339	0.631–2.840	0.4465
Financial situation ^6^	1.210	0.601–2.434	0.5936
Formerly smoking ^7^	0.685	0.374–1.254	0.2204
Currently smoking ^7^	0.674	0.245–1.859	0.4464
Alcohol consumption ^7^	1.000	0.481–2.078	1.0000
Antihypertensive drugs ^7^	1.250	0.649–2.407	0.5051
Hypolipemic drugs ^7^	1.483	0.538–4.088	0.4464
Hypoglycaemic drugs ^7^	3.145	0.617–16.023	0.1679
Hypothyroidism drugs ^7^	0.660	0.295–1.478	0.3123

^1^ Women vs. men; ^2^ village vs. city; ^3^ in relationship vs. single; ^4^ high vs. other; ^5^ employed vs. other; ^6^ very good + good vs. other; ^7^ yes vs. no. ALMI—appendicular lean mass index; BMI—body mass index; BPA—bisphenol A; CI—confidence interval; HAM-D—Hamilton depression rating scale; HDL-C—high-density lipoprotein cholesterol; hsCRP—high-sensitivity C reactive protein; LDL—low-density lipoprotein cholesterol; LMI—lean mass index; MCI—mild cognitive impairment; NCF—normal cognitive function; OR—odds ratio; TC—total cholesterol; TG—triglycerides.

**Table 6:** Adjusted results of logistic regression analysis predicting the probability of MCI.

	OR	95% CI	*p*
Education ^1^	0.478	0.217–1.053	0.0220
Total physical activity [min/day]	1.000	0.999–1.000	0.0501

^1^ High vs. other. CI—confidence interval; MCI—mild cognitive impairment; OR—odds ratio.

## Data Availability

The data presented in this study are available on request from the corresponding author (J.W.).

## Supplementary Materials

The following supporting information can be downloaded at https://www.mdpi.com/article/10.3390/metabo14050271/s1: Table S1: STROBE statement checklist. Table S2: Comparison of study population according to urine BPA [µg/g creatinine] tertiles.
