# Supplementary material for: No Differences in Urine Bisphenol A Concentrations between Subjects Categorized with Normal Cognitive Function and Mild Cognitive Impairment Based on Montreal Cognitive Assessment Scores
Source: Metabolites. 2024 May 8;14(5):271. doi: 10.3390/metabo14050271 (PMC11123393; doi:10.3390/metabo14050271)
Supplement: Supplementary file 1 [file metabolites-14-00271-s001.zip › Supplementary Materials_5.05.2024_final.pdf]

Table SI. STROBE Statement—checklist of items that should be included in reports of observational studies

	Item No.	Recommendation	Page No.	Relevant text from manuscript
**Title and abstract**	1	(*a*) Indicate the study's design with a commonly used term in the title or the abstract	1	Therefore, this observational study aimed to compare urine BPA levels in subjects with normal cognitive function (NCF) and mild cognitive impairment (MCI).
		(*b*) Provide in the abstract an informative and balanced summary of what was done and what was found	1	A link between bisphenol A (BPA) exposure and cognitive disorders has been suggested. However, the differences in BPA concentrations between subjects with and without cognitive impairment have not been analysed. Therefore, this observational study aimed to compare urine BPA levels in subjects with normal cognitive function (NCF) and mild cognitive impairment (MCI). A total of 89 MCI subjects and 89 well-matched NCF individuals were included in this study. Cog-nitive functions were assessed using the Montreal Cognitive Assessment (MOCA) scale. Urine BPA concentrations were evaluated by gas chromatography-mass spectrometry and adjusted for creatinine levels. Moreover, anthropometric parameters, body composition, sociodemographic factors and physical activity were also assessed. Creatinine-adjusted urine BPA levels did not differ between the NCF and MCI groups (1.8 (1.4 – 2.7) vs. 2.2 (1.4 – 3.6) µg/g creatinine, p = 0.1528). However, there were significant differences in MOCA results between groups when the study population was divided into tertiles according to BPA concentrations (p = 0.0325). Nevertheless, multivariate logistic regression demonstrated that only education levels were independently associated with MCI. In conclusion, urine BPA levels are not significantly different be-tween subjects with MCI or NCF individuals but these findings need to be confirmed in further studies.
**Introduction**				
Background/rationale	2	Explain the scientific background and rationale for the investigation being reported	1-2	The global elderly population is projected to increase to 1.4 billion by 2030, 2.1 bil-lion by 2050, and 3.1 billion by 2100 [1]. A significant repercussion of ageing is the on-set of mild cognitive impairment (MCI), a condition prevalent in about 20% of individuals over 50 [2]. MCI manifests as a decline in cognitive abilities, including memory, orientation, planning, decision-making, and comprehension, but does not significantly impact daily social or occupational functions [3,4]. It can affect language, visuospatial skills, attention, and executive functions, sometimes involving memory loss [5]. The condition is associated with an increased risk of developing dementia, with an estimated 5% to 10% of MCI patients progressing to dementia annually [6]. Prompt diagnosis could help in slowing the advancement of the disease, so identifying risk factors leading to MCI and its evolution into more severe neurocognitive disorders is crucial. Research has pinpointed several causative factors such as inadequate physical activity, alcohol consumption, smoking, substandard diet, social,

				environmental, and others [7]. Moreover, it was suggested that exposure to bisphenol A (BPA) may affect cognitive functions [8].<br>BPA (4,4-(propane-2-ylidene)diphenol) is widely utilised to produce polycarbonate plastics and epoxy resins, appearing in various consumer products, including food containers, packaging materials, cans, bottles and plastic bags. Dietary sources are typically the primary route of BPA exposure [9], with BPA being rapidly absorbed in the gastrointestinal tract and almost entirely metabolised in the liver through con-jugation with glucuronic acid and its remnants eliminated via urine in less than 24 hours. Thus, BPA exposure can be assessed in urine [10,11], with approximately 90% of urine samples testing positive for BPA [12]. As an endocrine-disrupting chemical, BPA's potential link to various health issues has been investigated [13–15], suggesting a correlation between BPA exposure and neurological disorders and cognitive impairment [16–18]. Animal studies showed that BPA could impair certain brain functions, including memory and learning [19–21]. Moreover, prenatal BPA exposure has been linked to neurobehavioral challenges in children [22,23], with maternal exposure affecting brain regions such as the postcentral gyrus, the opercular part of the inferior frontal gyrus, and the superior occipital gyrus [24]. However, there is a lack of studies assessing BPA levels in individuals with and without cognitive impairments.
Objectives	3	State specific objectives, including any prespecified hypotheses	2	Therefore, the purpose of the study was to assess urine bisphenol A levels in sub-jects with MCI and normal cognitive function (NCF).
**Methods**				
Study design	4	Present key elements of study design early in the paper	2	This observational study was approved by the Ethical Committee of the Poznan University of Medical Sciences (protocol no.: 249/22, date of approval: 10.03.2022). The study was performed according to the Strengthening the Reporting of Observational Studies in Epidemiology (STROBE) [25] and the Declaration of Helsinki [26]. All participants provided written informed consent. In this study, we employed a cross-sectional study design to compare individuals with MCI and NCF [27].
Setting	5	Describe the setting, locations, and relevant dates, including periods of recruitment, exposure, follow-up, and data collection	2	Recruitment and enrollment occurred over approximately 14 months (from July 2021 to August 2022) through the distribution of leaflets and posters, posting of recruitment information on the internet, and invitations to par-ticipate in the survey sent to various companies, associations and institutions located in Poznań (Greater Poland Voivodeship) and the surrounding area. The qualification visits were performed by physicians in the Department of Pediatric Gastroenterology and Metabolic Diseases, Poznan University of Medical Sciences.
Participants	6	(*a*) *Cohort study*—Give the eligibility criteria, and the sources and methods of selection of participants. Describe methods of follow-up	2	Participants were eligible if they were 50 to 70 years old and scored 19 to 26 points (the MCI group) or 27 to 30 points (the NCF group) on the Montreal Cognitive Assessment (MOCA) scale. Among the criteria for exclusion was a history of depression treatment, obtaining in the Hamilton Depression Rating scale (HAM-D)

		Case-control study—Give the eligibility criteria, and the sources and methods of case ascertainment and control selection. Give the rationale for the choice of cases and controls<br>*Cross-sectional study*—Give the eligibility criteria, and the sources and methods of selection of participants		test scores of more than 13 points, cognitive enhancement drug or psychotropic medication usage, significant alcohol consumption, substance abuse disorders, mental health conditions, Parkinson's disease, Alzheimer's disease, dementia, anaemia, diabetes of at least 10 years duration, severe chronic kidney and hepatic disorders, recent chemotherapy or radiotherapy for cancer within the past five years, stroke, seizures within the past two years, significant head injury, hypothyroidism with abnormal thyrotropic hormone levels, other severe chronic illnesses precluding participation, as well as conditions such as blindness, deafness, communication challenges, or other disabilities that might impede involvement in the study.
		(*b*) *Cohort study*—For matched studies, give matching criteria and number of exposed and unexposed<br>*Case-control study*—For matched studies, give matching criteria and the number of controls per case	4	Additionally, among the participants with MCI who were part of the randomised controlled trial [35] and collected a urine sample, 89 were selected to match the NCF group by age, sex, and BMI for inclusion in this analysis.
Variables	7	Clearly define all outcomes, exposures, predictors, potential confounders, and effect modifiers. Give diagnostic criteria, if applicable	2-4	2.3 Montreal Cognitive Assessment scale<br>The MOCA questionnaire was completed during the qualification visit to assign the participants to the MCI and NCF groups. The assessment was performed by physicians who obtained certificates for MOCA administration. A MOCA score < 19 points indicated dementia, 19-26 points were classified as MCI and scores > 26 points suggested NCF [28].<br>2.4 Hamilton Depression Rating scale<br>The HAM-D questionnaire was also applied to exclude the subjects with active depression. A HAM-D score ≥ 23 points indicated very severe depression, 18-22 points severe depression, 14-18 points moderate depression, 8-13 points mild depression and < 7 points no depressive symptoms [29,30].<br>2.5 International Physical Activity Questionnaires<br>The long Polish version of the International Physical Activity Questionnaire (IPAQ) was used to assess self-reported physical activity. This 27-item survey assessed the duration, frequency and intensity of physical activity performed within the last seven days. Four different types of physical activity were assessed: job- and transport-related, domestic and sedentary time on a usual weekday and weekend day. Only activities with a duration of at least ten minutes were registered to calculate total physical activity [31].<br>2.6 Anthropometric parameters and body composition<br>Trained personnel measured weight and height using the calibrated scale with a stadiometer (Radwag, WPT 100/200 OW, Radom, Poland) to calculate body mass index (BMI) [32]. The measurements were performed without shoes and in underwear. The waist circumference was measured at the umbilicus in the standing position on bare skin using non-stretchable tape (Seca, Hamburg, Germany) [33]. Body composition measurements were conducted through dual-energy X-ray absorptiometry (DEXA), utilizing the Hologic Discovery DEXA system (Bedford,

				MA, USA). The analysis focused on evaluating lean mass parameters, such as the appendicular lean mass index and the lean mass index[34].<br>2.7 Sociodemographic questionnaire<br>The subjects' place of residence, relationship status, education level, socio-occupational status, financial situation, and smoking and alcohol habits, as well as the use of select-ed groups of medications, were evaluated using sociodemographic questionnaires.<br>2.8 Bisphenol A levels<br>The study participants were asked to provide a fasting urine sample, which was frozen at -80◦C and stored until analysis of creatinine and total BPA levels. Creatinine levels were assessed using enzymatic methods to adjust total BPA levels to creatinine in urine. Total (free plus conjugated species) BPA concentrations were measured on gas chromatography-mass spectrometry (GC-MS). Measurements were performed by an Agilent Technologies 7890A gas chromatography system connected to a 5975C VL MSD mass spectrometer with a three-axis detector (Agilent Technologies, Waldbronn, Germany). Briefly, 0.5 ml of the urine sample was mixed with 50 µl of internal standard (deuterated BPA-d16 at 500 ng/ml) and 30 µl of acetate buffer (pH=5.5) before the addition of 30 µl of β-glucuronidase/sulfatase (Helix pomatia, diluted 10 x to 100000 U/ml in acetate buffer). This mixture was incubated at 37°C for three hours, and BPA was extracted with 3 x 4 ml of dichloromethane : hexane (1:1) mixture. The extract was evaporated to dryness under a stream of nitrogen and silylated with the addition of 100 µl of N,O-bis(trimethylsilyl)trifluoroacetamide (BSTFA): pyridine (1:1) at 80°C for 30 minutes. The samples were placed in chromatographic vials with an insert for GC-MS analysis. All reagents and chemicals used to total BPA measured were pur-chased from the Merck Life Science Sp. z. o. o., Poland (an affiliate of Merck KGaA, Darmstadt, Germany). The following GC settings were used: oven temperature of 90°C for one minute, with an increase of 10°C/minute to 240°C, held for two minutes, then increased by 20°C/minute to 310°C, and held for three minutes. The carrier gas was helium maintained at a constant pressure mode with a flow rate of 1 ml/minute at 90°C. The HP-5MS column measured 30 m x 0.25 mm x 0.25 µm. The injector port temperature was set at 295°C in splitless mode. The MS detector used electron ionisation with the ion source temperature at 230°C and the quadrupole temperature at 150°C.
Data sources/ measurement	8*	For each variable of interest, give sources of data and details of methods of assessment (measurement). Describe comparability of assessment methods if there is more than one group	2-4	2.3 Montreal Cognitive Assessment scale<br>The MOCA questionnaire was completed during the qualification visit to assign the participants to the MCI and NCF groups. The assessment was performed by physi-cians who obtained certificates for MOCA administration. A MOCA score < 19 points indicated dementia, 19-26 points were classified as MCI and scores > 26 points suggested NCF [28].<br>2.4 Hamilton Depression Rating scale

The HAM-D questionnaire was also applied to exclude the subjects with active depression. A HAM-D score ≥ 23 points indicated very severe depression, 18-22 points severe depression, 14-18 points moderate depression, 8-13 points mild depression and < 7 points no depressive symptoms [29,30].

2.5 International Physical Activity Questionnaires

The long Polish version of the International Physical Activity Questionnaire (IPAQ) was used to assess self-reported physical activity. This 27-item survey assessed the duration, frequency and intensity of physical activity performed within the last seven days. Four different types of physical activity were assessed: job- and transport-related, domestic and sedentary time on a usual weekday and weekend day. Only activities with a duration of at least ten minutes were registered to calculate total physical activity [31].

2.6 Anthropometric parameters and body composition

Trained personnel measured weight and height using the calibrated scale with a stadiometer (Radwag, WPT 100/200 OW, Radom, Poland) to calculate body mass index (BMI) [32]. The measurements were performed without shoes and in underwear. The waist circumference was measured at the umbilicus in the standing position on bare skin using non-stretchable tape (Seca, Hamburg, Germany) [33]. Body composition measurements were conducted through dual-energy X-ray absorptiometry (DEXA), utilizing the Hologic Discovery DEXA system (Bedford, MA, USA). The analysis focused on evaluating lean mass parameters, such as the appendicular lean mass index and the lean mass index[34].

2.7 Sociodemographic questionnaire

The subjects' place of residence, relationship status, education level, socio-occupational status, financial situation, and smoking and alcohol habits, as well as the use of select-ed groups of medications, were evaluated using sociodemographic questionnaires.

2.8 Bisphenol A levels

The study participants were asked to provide a fasting urine sample, which was frozen at -80◦C and stored until analysis of creatinine and total BPA levels. Creatinine levels were assessed using enzymatic methods to adjust total BPA levels to creatinine in urine. Total (free plus conjugated species) BPA concentrations were measured on gas chromatography-mass spectrometry (GC-MS). Measurements were performed by an Agilent Technologies 7890A gas chromatography system connected to a 5975C VL MSD mass spectrometer with a three-axis detector (Agilent Technologies, Waldbronn, Germany). Briefly, 0.5 ml of the urine sample was mixed with 50 µl of internal standard (deuterated BPA-d16 at 500 ng/ml) and 30 µl of acetate buffer (pH=5.5) before the addition of 30 µl of β-glucuronidase/sulfatase (Helix pomatia, diluted 10 x to 100000 U/ml in acetate buffer). This mixture was incubated at 37°C for three hours, and BPA was extracted with 3 x 4 ml of dichloromethane : hexane (1:1) mixture. The extract was evaporated to dryness under a stream of nitrogen and silylated with the

				addition of 100 µl of N,O-bis(trimethylsilyl)trifluoroacetamide (BSTFA): pyridine (1:1) at 80°C for 30 minutes. The samples were placed in chromatographic vials with an insert for GC-MS analysis. All reagents and chemicals used to total BPA measured were pur-chased from the Merck Life Science Sp. z. o. o., Poland (an affiliate of Merck KGaA, Darmstadt, Germany). The following GC settings were used: oven temperature of 90°C for one minute, with an increase of 10°C/minute to 240°C, held for two minutes, then increased by 20°C/minute to 310°C, and held for three minutes. The carrier gas was helium maintained at a constant pressure mode with a flow rate of 1 ml/minute at 90°C. The HP-5MS column measured 30 m x 0.25 mm x 0.25 µm. The injector port temperature was set at 295°C in splitless mode. The MS detector used electron ionisation with the ion source temperature at 230°C and the quadrupole temperature at 150°C.
Bias	9	Describe any efforts to address potential sources of bias	4	Additionally, among the 198 participants with MCI who were part of the randomised controlled trial [35], 89 were selected to match the NCF group by age, sex, and BMI for inclusion in this analysis.
Study size	10	Explain how the study size was arrived at	4	The power calculation was performed using preliminary findings from a pilot study and G*Power 3.1 software from the University of Kiel, Germany. Assuming an expected dropout rate of 20%, enrolling 166 participants (83 in each group) would ensure the study has over 80% statistical power to identify a meaningful difference of 0.5 µg total urine BPA/g creatinine (assuming a standard deviation of 35% of the mean) between the groups at a significance level of 0.05.
Quantitative variables	11	Explain how quantitative variables were handled in the analyses. If applicable, describe which groupings were chosen and why	2,4	Participants were eligible if they were 50 to 70 years old and scored 19 to 26 points (the MCI group) or 27 to 30 points (the NCF group) on the Montreal Cognitive Assessment (MOCA) scale.<br>individuals. Moreover, the Kruskal-Wallis test and the Jonckheere-Terpstra trend test were performed to compare subjects divided into tertiles according to urine BPA levels.
Statistical methods	12	(*a*) Describe all statistical methods, including those used to control for confounding<br>(*b*) Describe any methods used to examine subgroups and interactions	4	Statistical analyses were calculated using Statistica 13.0 (TIBCO Software Inc., Palo Alto, CA, USA) and PQstat 1.8.4 (PQStst Software Poznań/Plewiska, Poland) software. The Shapiro-Wilk test was used to check if the data were normally distributed. The Mann-Whitney test was applied to compare MCI and NCF individuals. More-over, the Kruskal-Wallis test and the Jonckheere-Terpstra trend test were performed to compare subjects divided into tertiles according to urine BPA levels. The Fisher exact test, Pearson's Chi-squared test and the Cochran-Armitage trend test were applied to evaluate categorical variables. In addition, logistic regression was performed to deter-mine the variables predicting the occurrence of MCI, while linear regression analysis was performed to analyse the relationship between urine BPA concentration and selected variables. The variables from the univariate regression analysis with a significance level of $p < 0.1$ were included in a multivariate regression model. The propensity score matching 1:1 method was used to match subjects from the MCI group with those from the NCF group based on age, sex, and

				BMI. All data are presented as medians and interquartile ranges or as counts and percentages. Analysis was used at an α-level of 0.05 to identify statistical significance.
		(*c*) Explain how missing data were addressed	5	See Figure 1
		(*d*) *Cohort study*—If applicable, explain how loss to follow-up was addressed<br>*Case-control study*—If applicable, explain how matching of cases and controls was addressed<br>*Cross-sectional study*—If applicable, describe analytical methods taking account of sampling strategy	4	Figure 1 illustrates the workflow of the study. As described earlier [27], approximately 1,000 individuals were interested in participating, of which 969 were eligible. Of these, 99 were initially placed in the NCF group, but one participant opted out of the study and nine were excluded from the analysis due to various reasons, so the NCF group ultimately included 89 participants. Additionally, among the 198 participants with MCI who were part of the randomised controlled trial [35], 89 were selected to match the NCF group by age, sex, and BMI for inclusion in this analysis. The baseline characteristics of the participants, such as sex, age, body measurements, HAM-D score, total physical activity, and socio-demographic aspects, are detailed in Tables 1 and 2, showing no significant differences between the groups.
		(*e*) Describe any sensitivity analyses	-	-
**Results**				
Participants	13*	(a) Report numbers of individuals at each stage of study—eg numbers potentially eligible, examined for eligibility, confirmed eligible, included in the study, completing follow-up, and analysed	4	Figure 1 illustrates the workflow of the study. As described earlier [27], approximately 1,000 individuals were interested in participating, of which 969 were eligible. Of these, 99 were initially placed in the NCF group, but one participant opted out of the study and nine were excluded from the analysis due to various reasons, so the NCF group ultimately included 89 participants. Additionally, among the 198 participants with MCI who were part of the randomised controlled trial [35], and collected a urine sample, 89 were selected to match the NCF group by age, sex, and BMI for inclusion in this analysis. The baseline characteristics of the participants, such as sex, age, body measurements, HAM-D score, total physical activity, and socio-demographic aspects, are detailed in Tables 1 and 2, showing no significant differences between the groups.
		(b) Give reasons for non-participation at each stage		
		(c) Consider use of a flow diagram	5	Figure 1
Descriptive data	14*	(a) Give characteristics of study participants (eg demographic, clinical, social) and information on exposures and potential confounders	4	The baseline characteristics of the participants, such as sex, age, body measurements, HAM-D score, total physical activity, and socio-demographic aspects, are detailed in Tables 1 and 2, showing no significant differences between the groups.
		(b) Indicate number of participants with missing data for each variable of interest	5-6	Tables 1-2
		(c) *Cohort study*—Summarise follow-up time (eg, average and total amount)	-	-
Outcome data	15*	*Cohort study*—Report numbers of outcome events or summary measures over time	-	-

		Case-control study—Report numbers in each exposure category, or summary measures of exposure	-	-
		Cross-sectional study—Report numbers of outcome events or summary measures	-	-
Main results	16	(*a*) Give unadjusted estimates and, if applicable, confounder-adjusted estimates and their precision (eg, 95% confidence interval). Make clear which confounders were adjusted for and why they were included	7-8	Figure 2, Figure 3
		(*b*) Report category boundaries when continuous variables were categorized	2,4	Participants were eligible if they were 50 to 70 years old and scored 19 to 26 points (the MCI group) or 27 to 30 points (the NCF group) on the Montreal Cognitive Assessment (MOCA) scale.<br>individuals. Moreover, the Kruskal-Wallis test and the Jonckheere-Terpstra trend test were performed to compare subjects divided into tertiles according to urine BPA levels.
		(*c*) If relevant, consider translating estimates of relative risk into absolute risk for a meaningful time period	-	-
Other analyses	17	Report other analyses done—eg analyses of subgroups and interactions, and sensitivity analyses	8-10	Tables 3-6
**Discussion**				
Key results	18	Summarise key results with reference to study objectives	11	The present study revealed no significant difference in creatinine-adjusted urine BPA levels between subjects with NCF and MCI, suggesting that BPA exposure may not directly differentiate these groups. However, when the study population was divided into tertiles according to BPA concentrations, there were significant differences between MOCA scores.
Limitations	19	Discuss limitations of the study, taking into account sources of potential bias or imprecision. Discuss both direction and magnitude of any potential bias	12	However, this study has some limitations, including categorising participants into the MCI and NCF groups based solely on MOCA test results. Moreover, we categorised participants into groups based on a single cognitive function assessment and did not compare it against peers of the same sex, age, and education levels. Since the MOCA assessment was also not repeated for study participants and we did not evaluate symptoms of subjective cognitive decline, there could potentially be a risk of inappropriately categorising subjects into study groups. It should also be highlighted that most individuals included in our study had higher education levels, lived in the city, were professionally active, and had moderately good health status. Therefore, our results should be generalised with caution to other populations, including subjects with lower education levels or those who live in rural environments, as BPA exposure in these individuals might differ. Another limitation of this study is relying on a single

				urine sample to measure BPA levels, which may not accurately reflect long-life exposure. Additionally, no assessment of BPA in blood samples was conducted. Furthermore, we did not evaluate BPA exposure directly and only measured total urine BPA levels without analysing the concentrations of free and conjugated BPA. Assessing conjugated BPA levels could mitigate potential BPA contamination during the collection, storage, and analysis of urine samples, as this form is produced within the human body. However, analysis of conjugated form may be difficult, and therefore, measurement of total BPA concentrations is recommended to assess BPA exposure [79]. Moreover, the hydration levels of the study participants were not assessed, while hydration might affect urine creatinine levels [80].
Interpretation	20	Give a cautious overall interpretation of results considering objectives, limitations, multiplicity of analyses, results from similar studies, and other relevant evidence	10-12	In our study, BPA was detected in all analysed samples with a median urine BPA concentration of 2.1 µg/g creatinine, which is slightly higher than previously reported [36–38]. Park et al. [38] reported a mean urine BPA concentration of 2.01 µg/g creatinine in 2044 Korean participants across different age groups, collecting 12-hour urine samples and using high-performance liquid chromatography-tandem mass spectrometry. They did not observe any differences between men and women but noted a positive association between BPA levels and age. The authors also performed a literature search to assess differences in urine BPA levels between different countries and found higher urine BPA levels in the European population than in people from other countries. The researchers speculated that these differences in BPA levels may result from variations in canned food intake worldwide [39]. Calafat et al. [36] analysed BPA concentrations in 394 adult participants of the Third National Health and Nutrition Examination Survey study using the isotope dilution GC-MS method and spot-urine samples collected at various times throughout the day. BPA levels were detected in 95% of the analysed samples, with a median BPA concentration of 1.32 µg/g creatinine. In addition, Koch et al. [37] investigated BPA levels in 24-hour urine and plasma samples from the German Environmental Specimen Bank spanning from 1995 to 2009 by high-performance liquid chromatography coupled to isotope dilution tandem mass spectrometry methods, and BPA was present in more than 96% of samples. The average total BPA concentration was slightly lower than observed in our study (1.81 µg/g creatinine). Also, a recent meta-analysis, which included 15 studies and 28,353 individuals, reported that BPA was present in 90% of samples with pooled BPA concentrations of 1.76 µg/g creatinine. Moreover, this meta-analysis showed that factors such as age, sex, residence of study participants and the measurement method did not affect urine BPA levels [40]. In our study, univariate linear regression showed that age and socio-occupational status were associated with urine BPA concentrations. However, these factors were not associated with creatinine-adjusted urine BPA concentrations in multivariate analysis. Nevertheless, it was previously suggested that men might be more sensitive to BPA than women due to the greater binding to oestrogen receptors [41]. Besides, Zhan et al. [42] reported differences in urine BPA

levels between men and women and subjects of different ages. The predominance of women in our study population may partly explain why sex was not identified as a factor influencing BPA levels in our analysis. However, we speculate that different levels of BPA exposure from the diet and other sources might partly explain differences in urine BPA levels and factors affecting BPA concentrations observed in various studies [43].

The negative effect of BPA on human health is well known. Bao et al. [14] demonstrated that higher exposure to BPA may increase all-cause mortality in a US population. Moon et al. [44], in their meta-analysis, showed that urine BPA levels are associated with the prevalence of cardiovascular diseases. BPA exposure is also related to an increased risk of type 2 diabetes mellitus [45]. Moreover, there is a relationship between BPA concentrations and obesity [46], as well as polycystic ovary syndrome [47]. It has also been suggested that BPA exposure may affect cognitive function [8]. However, our study did not detect differences in creatinine-adjusted urine BPA levels between MCI and NCF subjects, which may be associated with a small sample size and measuring the BPA concentration only in a single urine sample. Nevertheless, subjects in tertile II of BPA levels had statistically significantly higher MOCA scores than subjects from the highest tertile.

Interestingly, our analysis revealed greater variability in BPA levels among participants diagnosed with MCI compared to those with NCF. Several factors could contribute to this variability, including the subjects' lifestyle behaviours and their health status. Differences in dietary habits [27] and physical activity levels [48] could account for this variability. Moreover, there may be physiological differences in how BPA is metabolised or excreted in individuals with MCI compared to those without cognitive impairments. Changes in kidney function, which are sometimes associated with cognitive decline, could also influence BPA levels [49].

This is the first study that compared BPA levels between NCF and MCI participants. Previous studies that assessed the effect of BPA on cognition focused on the assessment of the impact of maternal exposure on foetus neurodevelopment or evaluated the effect of BPA exposure in children [22,50,51]. Braun et al. [51] observed that prenatal BPA exposure had a more negative effect on cognitive function in boys than in girls. BPA concentrations affected behaviours involving internalisation and somatisation and were associated with poorer working memory. Similarly, Rodríguez-Carrillo et al. [50] showed that BPA concentrations standardised to creatinine levels were associated with a higher risk of poorer working memory scores in school-age boys. Huang et al. [22] discovered the different effects of maternal BPA exposure in girls and boys. Higher BPA levels in girls were associated with enhanced dangers of self-control inhibition, developing metacognition issues, behavioural challenges, difficulties in peer relationships, elevated total difficulties score and increased impact factor score, whereas higher prenatal BPA levels in boys increased the risk of behavioural problems. In addition, prenatal BPA exposure was also associated with the

prevalence of attention deficit hyperactivity disorder (ADHD) in children aged three years. An association between BPA levels and ADHD prevalence was also confirmed by Gok et al. [18], who reported that ADHD subjects had significantly higher BPA levels than healthy participants. However, in this study, BPA concentrations did not correlate with cognitive function.

Several mechanisms have been proposed to explain the potential negative impact of BPA on cognitive functions [52,53]. Both in vivo and in vitro studies have demonstrated the effect of BPA on apoptosis, oxidative stress, inflammation, mitochondrial dysfunction and endoplasmic reticulum stress [54–58]. Moreover, the detrimental effect of BPA on myelination processes and neuronal integrity has been reported in both cell and animal studies [59,60]. It has also been shown that BPA might modulate neurotransmitter levels [61–64]. Furthermore, BPA's neurotoxicity has been associated with inhibition of neurogenesis [65] and reduced synaptic plasticity in rats [66]. Additional research has identified decreased axon length in zebrafish models [57], microglial DNA damage, and astrogliosis as responses to BPA exposure in explant models [67]. Li et al. [68,69], in human and animal studies, suggested that BPA may disrupt the homeostasis of energy metabolism and insulin signalling pathways, thereby impairing cognitive functions. However, another in vitro study noted that BPA affects intracellular $Ca2+$ balance [70]. Besides, incubation of a human neuroblastoma cell line with BPA increased levels of β-amyloid and tau proteins, which are linked to the pathogenesis of Alzheimer's disease [71].

Herein, we also performed logistic regression to identify independent determinants of MCI prevalence. The univariate logistic regression revealed that total physical activity and education impact MCI development. However, despite the suggestion that physical activity may be a protective factor for MCI [72–74], the multivariate logistic regression analysis demonstrated that only lower education levels were associated with higher MCI prevalence. Our findings are in line with those previously reported [75–77]. Bai et al. [75] reported that MCI prevalence increased with lower education levels, while Xue et al. [76] found that higher education levels may predict revision from MCI to NCF. Moreover, Vadikolias et al. [77] indicated that education was associated with results obtained in denominating entities, definitions, linguistic constructs, ability to name upon confrontation or phonetic assistance. In addition, Xu et al. [78], in their meta-analysis, showed that the risk of dementia was lowered by 7% for each additional year of education.

Our results have some potential clinical implications. First, the finding that a higher level of education may reduce the risk of developing cognitive disorders indicates that subjects with lower educational levels could benefit from earlier and more frequent cognitive assessments to detect potential decline more promptly. Additionally, although physical activity was not independently associated with MCI in our multivariate regression analysis, its protective role in cognitive health, as confirmed in several studies [72-74], suggests that increasing physical activity could be a beneficial

				strategy to prevent cognitive decline. Furthermore, as the relationship between BPA and cognitive decline was not definitively established in our study, it is important to consider other factors that may contribute to the development of MCI.
Generalisability	21	Discuss the generalisability (external validity) of the study results	12	This study is among the initial investigations to compare urine BPA levels between individuals with NCF and MCI. The study's strengths include the implementation of stringent and well-defined criteria for inclusion and exclusion, along with the application of propensity score matching to ensure that both groups are comparable in terms of age, sex, and BMI, as well as the adjustment of urine BPA levels for creatinine concentrations.<br>It should also be highlighted that most individuals included in our study had higher education levels, lived in the city, were professionally active, and had moderately good health status. Therefore, our results cannot be generalised to other populations, including subjects with lower education levels or those who live in rural environments, as BPA exposure in these individuals might differ.
**Other information**				
Funding	22	Give the source of funding and the role of the funders for the present study and, if applicable, for the original study on which the present article is based	13	This research was funded by the National Science Center, grant number: UMO-2017/27/B/NZ7/02924. The APC was funded by the Department of Pediatric Gastroenterology and Metabolic Diseases, Poznan University of Medical Sciences.

*Give information separately for cases and controls in case-control studies and, if applicable, for exposed and unexposed groups in cohort and cross-sectional studies.

Note: An Explanation and Elaboration article discusses each checklist item and gives methodological background and published examples of transparent reporting. The STROBE checklist is best used in conjunction with this article (freely available on the Web sites of PLoS Medicine at http://www.plosmedicine.org/, Annals of Internal Medicine at http://www.annals.org/, and Epidemiology at http://www.epidem.com/). Information on the STROBE Initiative is available at www.strobe-statement.org.

Table S2. Comparison of study population according to urine BPA [µg/g creatinine] tertiles.

		I [< 1.506 µg/g creatinine] (*n* = 59)	II [1.506 – 2.655 µg/g creatinine] (*n* = 59)	III [> 2.655 µg/g creatinine] (*n* = 60)	*p*	*p* trend
		Median (Q1–Q3) / *n* (%)				
Age [years]		57 (53 – 61)	57 (53 – 62)	58 (55 – 64)	0.3015	0.1697
Weight [kg]		77.00 (67.35 – 86.80)	72.70 (64.30 – 88.30)	76.25 (60.85 – 84.55)	0.3381	0.1487
BMI [kg/m2]		27.51 (24.90 – 30.75)	27.12 (23.44 – 30.31)	27.01 (23.29 – 30.37)	0.5835	0.3737
Waist circumference [cm]		93 (83– 102)	90 (80 – 102)	90 (77 – 98)	0.7050	0.3924
LMI [kg/m^2]		16.40 (14.60 – 18.00)	15.50 (14.00 – 17.80)	15.65 (13.95 – 18.00)	0.1923	0.1339
ALMI [kg/m^2]		7.09 (6.18 – 7.97)	6.60 (5.76 – 7.63)	6.52 (5.66 – 7.85)	0.1001	0.0886
Total physical activity [min/day]		66 (47 – 123)	103 (58 – 163)	109 (66 – 152)	0.0951	0.0549
HAM-D [points]		3 (1 – 5)	3 (1 – 6)	3 (2 – 6)	0.5182	0.3338
Sex	Women	37 (62.7%)	48 (81.4%)	45 (75.0%)	0.0677	0.1331
	Men	22 (37.3%)	11 (18.6%)	15 (25.0%)		
Place of residence	Village	10 (16.9%)	9 (15.2%)	10 (16.7%)	0.1607	-
	City < 50.000 inhabitants	5 (8.5%)	4 (6.8%)	5 (8.3%)		
	City of 50.000-500.000 inhabitants	10 (16.9%)	2 (3.4%)	3 (5.0%)		
	City > 500.000 inhabitants	34 (57.7%)	44 (74.6%)	42 (70.0%)		
Relationship status	Formal/informal relationship	45 (76.3%)	47 (79.7%)	45 (75.0%)	0.7123	0.8653
	Single	14 (23.7%)	11 (18.6%)	15 (25.0%)		
	No information	0 (0.0%)	1 (1.7%)	0 (0.0%)		
Education	Primary	0 (0.0%)	0 (0.0%)	2 (3.3%)	0.2130	-
	Vocational	4 (6.8%)	0 (0.0%)	2 (3.3%)		
	Secondary	10 (16.9%)	9 (15.2%)	10 (16.7%)		
	High	45 (76.3%)	50 (84.8%)	46 (76.7%)		
Socio-occupational status	Employed	48 (81.4%)	49 (83.0%)	47 (78.3%)	0.3030	-
	Unemployed	2 (3.4%)	0 (0.0%)	0 (0.0%)		
	Pensioner	9 (15.2%)	10 (17.0%)	13 (21.7%)		
Financial situation	Very good	6 (10.2%)	5 (8.5%)	5 (8.3%)	0.9667	-

	Good	39 (66.1%)	40 (67.8%)	42 (70.0%)		
	Mediocre	14 (23.7%)	13 (22.0%)	12 (20.0%)		
	Bad	0 (0.0%)	1 (1.7%)	1 (1.7%)		
Formerly smoking	Yes	25 (42.4%)	25 (42.4%)	20 (33.3%)	0.5061	0.3342
	No	34 (57.6%)	34 (57.6%)	40 (66.7 %)		
Currently smoking	Yes	7 (11.9%)	6 (10.2%)	4 (6.7%)	0.6158	0.3115
	No	52 (88.1%)	53 (89.8%)	58 (93.3%)		
Alcohol consumption	Once a day	2 (3.4%)	2 (3.4%)	0 (0.0%)	0.7869	-
	Several times a week	7 (11.9%)	8 (13.6%)	7 (11.7%)		
	Once a week	11 (18.6%)	17 (28.8%)	17 (28.3%)		
	1-3 times a month	27 (45.8%)	20 (33.9%)	24 (40.0%)		
	Never	12 (20.3%)	12 (20.3%)	12 (20.0%)		
Antihypertensive drugs	Yes	19 (32.2%)	16 (27.1%)	15 (25.0%)	0.6685	0.3826
	No	40 (67.8%)	43 (72.9%)	45 (75.0%)		
Hypolipemic drugs	Yes	9 (15.2%)	5 (8.5%)	3 (5.0%)	0.1542	0.0572
	No	50 (84.8%)	54 (91.5%)	57 (95.0%)		
Hypoglycaemic drugs	Yes	4 (6.8%)	1 (1.7%)	3 (5.0%)	0.4003	0.6438
	No	55 (93.2%)	58 (98.3%)	57 (95.0%)		
Hypothyroidism drugs	Yes	11 (18.6%)	7 (11.9%)	11 (18.3%)	0.5297	0.9677
	No	48 (81.4%)	52 (88.1%)	49 (81.7%)		

ALMI – appendicular lean mass index; BMI – body mass index; BPA – bisphenol A; HAM-D – Hamilton depression rating scale; HDL-C – high-density lipoprotein cholesterol; hsCRP – high-sensitivity C reactive protein; LDL – low density lipoprotein cholesterol; LMI – lean mass index; MOCA – Montreal cognitive assessment scale; TC – total cholesterol; TG – triglycerides
